# Hypoxia-Regulated Tumor-Derived Exosomes and Tumor Progression: A Focus on Immune Evasion

**DOI:** 10.3390/ijms231911789

**Published:** 2022-10-04

**Authors:** Xuejun Shao, Shenghao Hua, Tao Feng, Dickson Kofi Wiredu Ocansey, Lei Yin

**Affiliations:** Clinical Laboratory, Children’s Hospital of Soochow University, Suzhou 215000, China

**Keywords:** hypoxia, exosome, immune evasion, surface recognition molecule, immune cell, metastasis, angiogenesis, therapy resistance

## Abstract

Tumor cells express a high quantity of exosomes packaged with unique cargos under hypoxia, an important characteristic feature in solid tumors. These hypoxic tumor-derived exosomes are, crucially, involved in the interaction of cancer cells with their microenvironment, facilitating not only immune evasion, but increased cell growth and survival, enhanced angiogenesis, epithelial–mesenchymal transition (EMT), therapeutic resistance, autophagy, pre-metastasis, and metastasis. This paper explores the tumor microenvironment (TME) remodeling effects of hypoxic tumor-derived exosome towards facilitating the tumor progression process, particularly, the modulatory role of these factors on tumor cell immune evasion through suppression of immune cells, expression of surface recognition molecules, and secretion of antitumor soluble factor. Tumor-expressed exosomes educate immune effector cells, including macrophages, monocytes, T cells, natural killer (NK) cells, dendritic cells (DCs), γδ T lymphocytes, regulatory T cells (Tregs), myeloid-derived suppressor cells (MDSCs), mast cells, and B cells, within the hypoxic TME through the release of factors that regulate their recruitment, phenotype, and function. Thus, both hypoxia and tumor-derived exosomes modulate immune cells, growth factors, cytokines, receptor molecules, and other soluble factors, which, together, collaborate to form the immune-suppressive milieu of the tumor environment. Exploring the contribution of exosomal cargos, such as RNAs and proteins, as indispensable players in the cross-talk within the hypoxic tumor microenvironmental provides a potential target for antitumor immunity or subverting immune evasion and enhancing tumor therapies.

## 1. Background

The hypoxic TME is a general characteristic of solid carcinoma and is associated with tumor aggressiveness and poor patient outcome [1]. Hypoxia induces the expression of a variety of genes linked with cell survival, metabolism, proliferation, apoptosis, angiogenesis, stemness, and EMT in a hypoxia-inducible factor 1 (HIF-1)-dependent or -independent manner [2,3,4,5]. In addition to its prediction of poor prognosis across all cancers [6], tumor hypoxia also serves as a center for the recruitment, polarization, and expansion of tumor-friendly stromal cell populations [7,8,9], where increasing hypoxia strongly correlates with a more aggressive cancer phenotype [10,11]. Within the hypoxic zones of the TME, cancer cells capably adapt and induce cell growth and proliferation by producing a variety of metabolic intermediates that function as precursors for biosynthetic pathways. The oxygen-deprived conditions facilitate the reduction in activation levels of tumor-infiltrating or cytotoxic cells, such as lymphocytes, CD8(+) T cells, and NK cells, while increasing tumor activity suppressive cells, such as MDSCs, Tregs, and their associated cytokines, results in tumor evasion of immune detection [8,12,13]. Moreover, antigen-presenting cells, including DCs, monocytes, and macrophages, are rendered less effective in recognizing and processing tumor antigens to activate host immune responses via polarization to tumor immune evasion phenotypes.

Extracellular vesicles, which include exosomes, apoptotic bodies, microvesicles, and oncosomes, refer to a heterogeneous population of lipid bilayer-enclosed vesicular bodies of cellular origin released by all eukaryote and prokaryote cells. While extracellular vesicles, in general, have gained increased traction in research, the role of exosomes in immune response across non-malignant and malignant disease progression has been well explored [14,15]. Within the hypoxic environment, varied cell types of the TME, such as cancer cells, immune cells, and fibroblasts, secrete exosomes that, together, contribute to modulating the host’s immunity. Tumor-derived exosomes are key mediators of the communication between tumor cells and or within the microenvironment [16,17]. It is asserted that tumor cells secrete a large number of exosomes that convey a variety of immunoregulatory cargos, including nucleic acids, proteins, and lipids, which assists in overcoming the hostile hypoxic microenvironment of solid tumors, leading to facilitated tumor development and metastasis [18]. It is well established that hypoxia within the TME influences the functions of immune cells through multiple overlapping mechanisms. Each cell within the TME, regardless of its transformation status, is capable of adapting to the hostile TME and expressing immunomodulatory signals or mediators, that, in turn, affect their functions directly or via the activation of other cells present in the tumor site [19]. Available data indicate that hypoxia promotes exosome secretion by cancer cells and considerably alters exosomal cargo [20,21], while exosomes, in turn, regulate the TME and enhance hypoxia [22,23]. In effect, the hypoxic tumor-derived exosome remodels the TME that facilitates the process of tumor cell growth and survival, EMT, angiogenesis, pre-metastasis, metastasis, therapy resistance, and evasion of immune surveillance. This paper reviews the modulatory role of hypoxia-induced tumor-derived exosomes toward tumor progression, with emphasis on the mechanisms of immune evasion via immune cell suppression, expression of surface molecules, and secretion of tumor-promoting soluble factors.

## 2. Overview of Hypoxic and Tumor-Derived Exosomal Effects on Tumor Progression

A developing tumor rapidly outgrows its oxygen demands and becomes subjected to hypoxia, which triggers the increased secretion of tumor-derived exosomes that enhance tumor immune evasion, EMT, and angiogenesis, hence, increasing tumor growth, migration, and metastasis. For instance, hypoxic tumor cells produce exosomes that are highly enriched in immunoregulatory cargos, including proteins and chemokines, such as colony-stimulating factor 1 (CSF-1), chemokine (C-C motif) ligand 2 (CCL2), ferritin H, ferritin L, and transforming growth factor β (TGFβ) [24]. The expression of microRNA (miR)-301a-3p by gastric cancer exosomes in an HIF-1α-dependent manner, results in its transmission between gastric cancer cells via exosomes to inhibit HIF-1α degradation by targeting prolyl hydroxylase 3 (PHD3), an essential hypoxia regulator capable of hydroxylating HIF-1α subunits to ubiquitinate degradation. This synergistic positive feedback loop facilitates tumor proliferation, migration, invasion, and EMT [25]. Moreover, the hypoxic microenvironment stimulates tumor cells to secrete miR-21-rich exosomes that are delivered to normoxic cells to promote prometastatic properties, including cell migration, invasion, and lymph node metastasis [26].

### 2.1. Angiogenesis

The hypoxic TME, as a key feature of solid tumors, induces cancer cells to produce exosomes and encourages angiogenesis, the formation of new blood vessels form from pre-existing vessels [27]. The detailed functions of hypoxic exosomes and the mechanisms underlying their effects continue to be explored across various TMEs [28,29]. Pancreatic cancer cell-derived hypoxic exosomes promote cell migration and tube formation of human umbilical vein endothelial cells (HUVECs) through the long noncoding RNA (lncRNA) UCA1, which acts as a sponge for miR-96-5p, impeding the inhibitory functions of miR-96-5p on the expression of its target gene AMOTL2 (angiomotin-like protein 2). In effect, hypoxic exosomal expressed UCA1 promotes angiogenesis and tumor growth via the miR-96-5p/AMOTL2/ERK1/2 axis [30]. A similar study demonstrated that hypoxic bladder cancer cells remodel the TME to enhance tumor growth and development by expressing the oncogenic long noncoding RNA (lncRNA)-UCA1-enriched exosomes, which also appears as a possible diagnostic biomarker in human serum for bladder cancer [18]. Other studies include hypoxic exosomal facilitation of angiogenesis and metastasis via altering the phenotype and transcriptome of endothelial cells [31] and hypoxic lung cancer-secreted exosomal miR-23a increase in angiogenesis and vascular permeability via targeting prolyl hydroxylase and tight junction protein ZO-1 [32].

### 2.2. Metastasis and Pre-Metastasis

While a pre-metastatic niche refers to the microenvironment, which is well prepared for tumor cells to colonize and disseminate to distant organs, metastasis is the spread of tumor cells from the point of origin to other parts of the body. These activities are the main cause of cancer-related mortality and morbidity across many types of cancers, accounting for approximately 90% of cancer deaths [33]. The hypoxic TME induces the expression of exosomal cargos that promote both the pre-metastatic and metastatic processes. The pre-metastatic niche, remolded by primary tumor-derived factors, such as exosomes and host stromal cells, contribute to cancer metastasis. Tumor-derived exosomes are capable of stimulating bone marrow-derived cell mobilization to form a pre-metastatic niche and even determine organotropic metastasis through the expression of integrins on their membrane [34]. The exosomal LncRNA BCRT1 is significantly upregulated in breast cancer tissues and positively correlates with tumor growth, metastasis, and poor prognosis in breast cancer patients by functioning through the HIF-1α/lncRNA BCRT1/miR-1303/PTBP3 pathways [35]. The exosome-mediated transfer of miR-193a-3p, miR-210-3p, and miR-5100 could also enhance lung cancer cell metastasis by activating STAT3 signaling-induced EMT [17]. Moreover, the transport of hypoxia-derived exosomal circ-133 into normoxic cancer cells promotes cell metastasis via the miR-133a/GEF-H1/RhoA axis [36].

### 2.3. EMT

This is a process where epithelial cells are transcriptionally reprogrammed, causing reduced adhesion and improved migration and invasion [3]. The hypoxic microenvironment gives rise to altered cellular metabolism and triggers varied molecular responses that contribute to triggering EMT, a process highly implicated in cancer metastasis and progression [37]. Exosomes obtained from hypoxic lung adenocarcinoma cells improve the migration, invasion, and proliferation capacity of normoxic lung adenocarcinoma cells via circSETDB1, which is significantly upregulated in hypoxia-induced exosomes compared with exosomes in the normal condition. Further analysis implicated the circSETDB1/miR-7/specificity protein 1 (Sp1) axis in the development and epithelial–mesenchymal transition (EMT) of lung adenocarcinoma [38]. Hypoxic-induced exosomal delivery of TGF-β contributes to the elevation of TGF-β signaling, resulting in the regulation of human MENA alternative splicing and promoting EMT in breast cancer via the TGF-β-RBFOX2 (RNA binding Fox-1 homolog 2)-ESRP1 (epithelial splicing regulatory protein 1) axis [39].

### 2.4. Autophagy

Autophagy is a highly conserved and regulated process that targets proteins and damaged organelles for lysosomal degradation to maintain cell metabolism, genomic integrity, and cell survival. However, autophagy plays a complicated role in tumorigenesis and therapy responsiveness, in that, while it suppresses tumor initiation in normal tissue, some tumors rely on it for maintenance and promotion [40,41]. Tumor-associated autophagy activation is one of the ways by which cancer cells survive hypoxia and collaborate with tumor-derived exosomes to contribute to HIF-1-mediated immune evasion [22]. In hepatocellular carcinoma, hypoxia-induced autophagy is associated with tumor growth, metastasis, and poor prognosis [42]. The detection of autophagy protein, microtubule-associated protein-1 light chain-3B in exosomes from advanced solid tumors, serves as a dynamic approach to monitor autophagy and could be utilized to study the effects of anti-neoplastic agents on autophagy and mechanisms of drug resistance [43]. It is also reported that key pathological pathways associated with esophageal squamous carcinoma include autophagy and cancer-associated pathways, among others [44].

### 2.5. Cell Survival and Proliferation

Hypoxic exosomes expressed by various cancer cells significantly upregulate the proliferation of endothelial cells as well as tumor cells, promoting tumor growth via the target of several signaling pathways, including the HIF1α signaling. Hypoxic conditions induce elevated secretion of exosomes by hepatocellular carcinoma cells and these exosomes, in turn, enhance the proliferation, migration, invasiveness, and EMT under normoxic conditions via miR-1273f transfer [45]. Similarly, hypoxia-induced exosome enriches in regulatory mRNAs and proteins, such as matrix metalloproteinases, IL-8, PDGFs, caveolin 1, and lysyl oxidase, mediate hypoxia-dependent intercellular signaling of the highly malignant brain tumor glioblastoma multiforme, promoting tumor cell proliferation and development [46]. Cancer-associated fibroblast-derived exosomes regulate the survival and proliferation of cancer cells. These exosomes increase the chemoresistance-inducing factor, Snail, in recipient epithelial cells and promote proliferation and drug resistance [47]. Extracellular vesicles expressed by metastatic hepatocellular carcinoma cells contain a significant quantity of the protein, complement factor H, which enhances tumor cell growth, migration, invasiveness, and liver tumor formation in mice [48]. Furthermore, hypoxic exosomes derived from bladder cancer cells promote cell proliferation, migration, and invasion, and the hypoxic exosomal lncRNA-UCA1 could be internalized by bladder cancer cells to mediate tumor growth and development [18].

### 2.6. Therapy Resistance

Hypoxic tumor-derived exosomes contribute to the formation and progression of the cancer processes, including various antitumor therapeutic resistance, such as chemoresistance [49], immune therapy resistance [50,51], and radiotherapy resistance [52,53]. Tumor hypoxia and aerobic glycolysis are well-known resistance factors for anticancer therapies. Tumor-associated macrophages increase AMP-activated protein kinase (AMPK) and peroxisome proliferator-activated receptor-gamma coactivator 1α (PPARγC1α) in the macrophages to facilitate tumor hypoxia and produce tumor necrosis factor (TNF)α to enhance tumor cell glycolysis. The resultant effect is a reduced response to anticancer therapies, including immunotherapy [54]. Tumor-associated macrophages have been identified as the primary source of programmed death-ligand 1 (PD-L1) in human and murine cholangiocarcinoma, where the PD-L1+ macrophages facilitate tumor progression. The authors noticed that immune checkpoint blockade of the tumor-associated macrophages did not reduce tumor progression due to a compensatory emergence and recruitment of granulocytic MDSCs that mediated immune escape by impairing T cell response [55].

### 2.7. Immune Evasion

**Figure 1:**
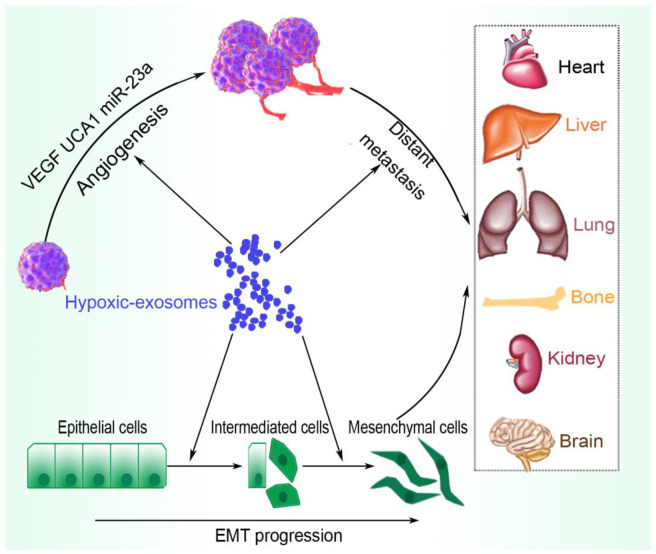
An overview of the promoting effects of the hypoxic-tumor-derived exosome. Tumor-derived exosomes transfer regulatory particles, such as VEGF, UCA1, and miR-23a, that induce angiogenesis and enhance tumor growth. The expressed exosomes also trigger EMT, which together contribute to enhanced metastasis.

Tumor immune evasion permits cancer cells to proliferate and metastasize and is associated with immunotherapy and radiotherapy failure, as well as chemoresistance. In the adaptive and innate immune systems, malignant tumor cells escape recognition and destruction via the HIF-1 in the hypoxic TME, where HIF-1 signaling suppresses immune attack by triggering increased expression of immune checkpoint molecules and tumor immune evasion factors, such as interleukin (IL)-10, vascular endothelial growth factor (VEGF), TGF-β, prostaglandin E2 (PGE2), and PDL-1/programmed death-1 (PD-1) [22]. Hypoxic tumor-derived exosomes suppress the immune system by regulating immune cells, such as macrophages, via the transfer of let-7a miRNA, which inhibits the insulin-Akt-mTOR (mammalian target of rapamycin) signaling pathway [24], promotes MDSCs via the miR-10a/Rora and miR-21/PTEN pathways [56], and impedes T-cell function via exosomal miR-24-3p-mediated target the fibroblast growth factors 11 (FGF 11) [57], among others, as detailed later in the review. Figure 1 summarizes the involvement of hypoxic tumor-derived exosomes in the induction of EMT and angiogenesis, enhancing metastasis to distant organs.

## 3. Hypoxia-Inducible Factor (HIF)

The central signaling associated with hypoxia is the HIF signaling pathway, a complex pathway and associated processes mediated by two major modulators that constitute a heterodimeric complex, which is the cytoplasmic oxygen-dependent HIF-α (HIF-1α, HIF-2α, and HIF-3α) and the constitutively expressed nuclear HIF-1β. The stabilization of the complex is facilitated by a group of oxygen- and iron-dependent enzymes called HIF-prolyl hydroxylase domain enzymes (PHD1-3) [58]. HIF plays an indispensable role in the hypoxic TME, where it not only participates in immune evasion but is involved in multiple aspects of tumor progression, such as cell proliferation, migration, EMT, metastasis, and angiogenesis [7,59]. Malignant tumor cells avoid recognition and destruction by both the innate and adaptive immune systems via the HIF-1, which is also associated with tumor cell immunotherapy resistance, radiotherapy resistance, and chemoresistance, enhancing cancer cells to proliferate and metastasize [60,61]. In these processes, hypoxia drives the stabilization of HIF, which acts as a central regulator to dampen antitumor immunity. The enhanced HIF signaling in cancer cells and immune cells promotes the recruitment and maintenance of pro-tumorigenic immune cells and inhibition of antitumorigenic activities, leading to immune evasion [22,62]. Moreover, the influence of HIF-1 on autophagy, immune checkpoint molecules, and immune suppression molecules, in addition to the potential effect of HIF-1 in the modulation of tumor-derived exosomes to assist tumor evasion, have been well described [22]. Based on its central role in immune evasion, targeting HIFs or their target gene products may restore antitumor immunity and enhance the response to immunotherapies.

Tumor cells are well known for shifting their metabolism to maintain high proliferation rates and survive in unfavorable environments with low oxygen and nutritional deficiency [63]. Several metabolic abnormalities in cancer cells elevate HIF-1 function, which serves as an important factor in the reprogramming of cancer metabolism through the stimulation of the transcription of genes that encode glycolytic enzymes and glucose transporters, which take up glucose and convert it to lactate. There is also the transcription of BNIP3, which activates selective mitochondrial autophagy and pyruvate dehydrogenase kinase 1, which shunts pyruvate away from the mitochondria. The resultant cancer metabolism shift promotes tumor progression [64]. For example, a shift in glutamine nitrogen metabolism participates in the malignant progression of cancer [65], while tumor stem cells also appear to adapt their metabolism to the alteration in the microenvironment by conveniently shifting energy production from one pathway to another or by acquiring intermediate metabolic phenotypes [66].

## 4. Hypoxia and Tumor-Derived Exosomal Components

**Figure 2:**
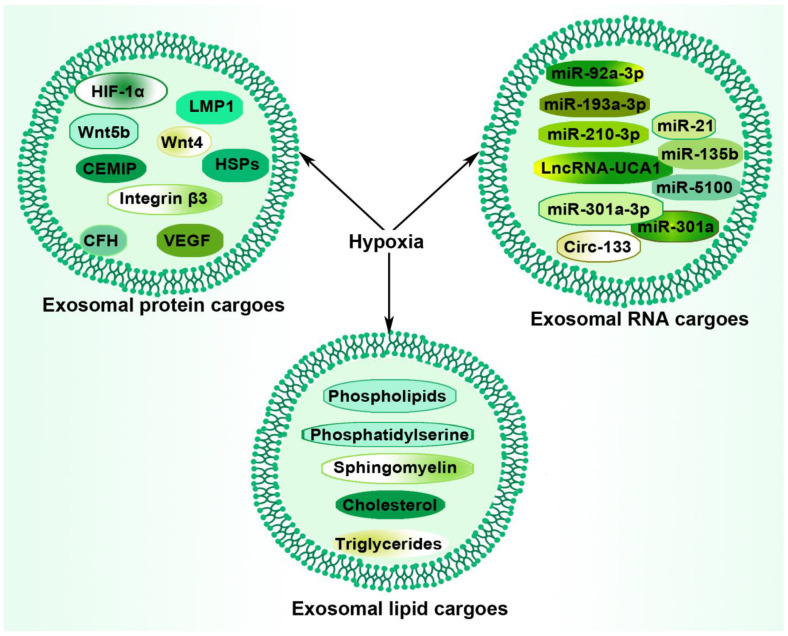
Hypoxia-induced packaging of exosomal cargos. Exosomes consist of three primary components: nucleic acids, proteins, and lipids. Hypoxia within the TME regulates the loading of these regulatory particles for subsequent transfer between cells, enhancing tumor promotion. Thus, these cargos could provide potential targets in the application of hypoxia tumor-derived exosomes in cancer therapeutics and diagnosis.

Exosomal cargos generally reflect the pathophysiological condition of the cell or microenvironment of origin. These cargos include all known constituents of a cell, including RNAs, DNAs, proteins, lipids, and metabolites, surrounded by a lipid bilayer [67]. It is reported that exosomes expressed by various types of tumor cells carry distinct molecular information from that produced by corresponding non-neoplastic or healthy cells and are capable of significantly modifying the TME and influencing tumor progression [68]. While the hypoxic condition within the TME stimulates exosome secretion from cancer cells to enhance local and distant communication [69,70], exosomes, in return, regulate hypoxia adaptation to facilitating the rebuilding of the microenvironment [1]. Within the hypoxic cancer microenvironment, the exosome-mediated multi-directional and mutual signal transmission among a variety of cell types facilitates immune evasion and tumor progression. A representative overview of hypoxia-induced packages of exosomal components is presented in Figure 2.

### 4.1. Hypoxia and Tumor-Derived Exosomal Protein

The hypoxic TME is associated with increased or altered exosomal protein content, helping in tumor immune evasion, progression, metastasis, and therapy resistance. Interestingly, the main mediator of hypoxia, HIF-1α, was found as an exosomal package in a transcriptionally active form in nasopharyngeal carcinoma cell-derived exosomes, where its expression promoted cancer invasive potential in association with latent membrane protein 1 (LMP1)-positive exosomes [71]. The exosomal package of HIF-1α for delivery within the hypoxic TME enhances tumor cells to evade degradation, as they utilize exosomes as a safe transporter of HIF-1α to the recipient cells, where it regulates its transcriptional targets and propagates hypoxic signaling. Hypoxic exosomes induce differential gene expression in recipient glioma cells, governed by the protein cargo being transferred via the exosomes [72]. Hypoxia stimulates tumor cells to release Wnt4-rich exosomes that are delivered to normoxic cells to enhance prometastatic behaviors, including enhanced tumor invasion and migration [73]. Moreover, several exosomal heat shock proteins (ex-HSPs), including HSP90 (α, β, Gp96, Trap1), HSP70, and large and small HSPs, have been identified as oncosomes (a type of extracellular vesicle) essential in the resistance-associated secretory phenotype (RASP) and can facilitate tumor progression and resistance against stressful conditions, such as hypoxia, drugs, radiation, and immune systems [74]. Other hypoxia-induced exosomal protein cargos include complement factor H [48], Integrin β3 [75], VEGF [23], and Wnt5b [76].

### 4.2. Hypoxia and Tumor-Derived Exosomal RNAs

Differential expression of circRNAs has been demonstrated between exosomes extracted from hypoxic and normoxic conditions. According to Pocock, hypoxic stress modulates the expression of a specific but expanding subset of miRNAs, termed hypoxamirs, while miRNAs, in turn, serve as ideal mediators of hypoxic stress responses, as they can modify gene expression both rapidly and reversibly [77]. A hypoxic-induced package of tumor-derived exosomal RNAs assists in all phases of tumor progression, including immune evasion. For instance, hypoxic exosomal miR-301a-3p induces the M2 polarization of macrophages in the pancreatic cancer microenvironment, leading to promoted malignant behaviors of the cancer cells [78]. Moreover, hypoxia promotes gastric cancer exosome secretion and increased exosomal miR-301a-3p expression in an HIF-1α-dependent manner, facilitating proliferation, migration, invasion, and EMT [25]. Exosome-mediated transfer of select miRNAs, including miR-210-3p, miR-193a-3p, and miR-5100, facilitates the invasion of lung cancer cells via the activation of STAT3 signaling-induced EMT [17]. The miR-21 is an oncogenic microRNA that modulates the expression of multiple cancer-related target genes, with its high stromal expression correlating with low E-cadherin expression and high metastasis-associated protein1 expression in colorectal cancer patients [79]. Hepatoma-derived exosomal miR92a-3p plays an important role in EMT progression and promotes metastasis by inhibiting PTEN and activating Akt/Snail signaling [80].

### 4.3. Hypoxia and Tumor-Derived Exosomal Lipids

Lipids are important components in exosomal membranes and are packaged into exosomes alongside nucleic acids and proteins. Regardless, far fewer data on their composition and biological effects are available compared to exosomal nucleic acids and proteins. Available studies indicate that the majority of the lipid classes in exosomes include phospholipids, sphingomyelin, cholesterol, and, to a lesser extent, triglycerides, suggesting that exosome membranes contain lipid raft-like domains [20]. A study reported that exosomal surface phosphatidylserine receptors, rather than CD29 and CD44, are responsible for the internalization of exosomes in the hypoxic microenvironment [81]. Tumor-derived exosomes carrying fatty acids induce dysfunctional DCs to promote immune evasion. Mechanistically, peroxisome proliferator-activated receptor (PPAR) α responds to the fatty acids delivered by tumor-derived exosomes, causing an excess lipid droplet biogenesis and elevated fatty acid oxidation, culminating in a metabolic shift toward mitochondrial oxidative phosphorylation, which, in turn, drives DC immune dysfunction [82]. Moreover, under hypoxia (1% O2), human prostate cancer cells and their expressed extracellular vesicles are highly enriched in triglycerides, owing to the stimulation of lipogenesis-related signaling molecules and enzymes. Interestingly, the inhibition of lipid utilization significantly compromised hypoxic tumor-cell proliferation and cyclooxygenase-2 (COX-2) inhibition also caused a significant reduction in cancer growth and invasiveness [83]. The increased lipid loading in tumor-derived exosomes could be a possible survival response to hypoxic stress as the stored lipids could aid tumor growth following reoxygenation. More importantly, exosomal lipids in the TME likely play important roles in immune evasion and tumor progression and, thus, it is worth further exploration. Table 1 summarizes the various exosomal components within the TME and their promoting effects on a variety of tumors.

## 5. Immune Cell Evasion

**Figure 3:**
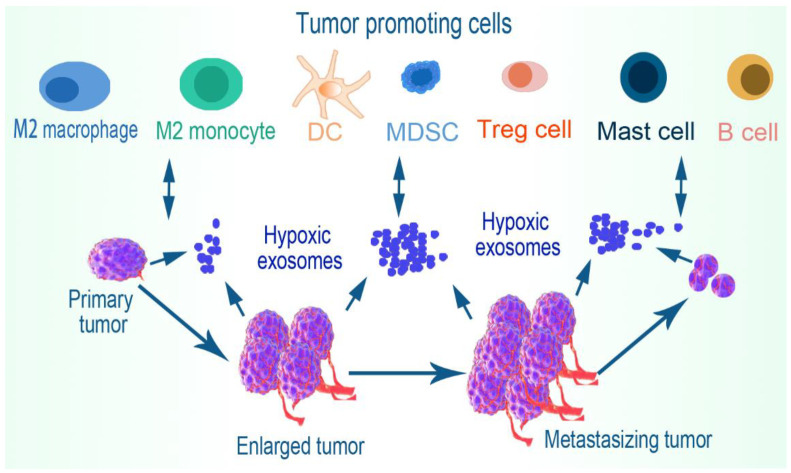
Tumor cell immune evasion. Within the TME, hypoxic exosomes induce the immune cells to become tumor friendly. Both the increased polarization of immune cells toward anti-inflammatory phenotypes and the deactivation/dysfunction of antitumor immune cells drive the tumor-promoting environment. The resultant microenvironment enhances tumor-friendly immune cell cytokine expression, together with other regulatory exosomal cargos that facilitate tumor cell immune evasion and result in tumor growth and metastasis.

Tumor-derived exosomes participate in the suppression of antitumor immunity in the hypoxic environment. Cancer cells transfer these extracellular vesicles in addition to a variety of secreted cytokines and receptors to host immune cells to impede both innate and adaptive immunity [86]. For instance, exosomes obtained from patients with neck and head cancers effectively induced CD8+ T cell apoptosis, upregulated Treg suppressor functions, and suppressed CD4+ T cell proliferation, in addition to the downregulation of NKG2D expression levels on NK cells [87,88]. Although the mechanisms underlying the observed immunostimulatory and immune evasion roles of tumor-derived exosomes are yet to be fully understood, hypoxic stress in the TME has been demonstrated to contribute to the variations in the exosomal constituents and consequent effects elicited [1,89,90]. In effect, hypoxic tumor-derived exosomes facilitate tumor progression by impeding cytotoxic immune cells, promoting the recruiting and activation of anti-inflammatory immune cells and switching cells toward tumor-promoting phenotypes, thus, facilitating tumor immune escape, growth, and metastasis (Figure 3).

### 5.1. Macrophages

A model study of exosome effects on tumor-infiltrating immune cells revealed a potent ability of hypoxia-induced vesicles to influence macrophage recruitment and promote M2-like polarization, both in vitro and in vivo. Additionally, compared to normoxia, hypoxic tumor exosomes improved oxidative phosphorylation in macrophages derived from bone marrow via the transfer of let-7a miRNA, causing inhibition of the insulin-Akt-mTOR signaling pathway. This finding demonstrates the hypoxic promoting effects on tumor secretion of biomolecule-loaded exosomes that capably modify the immune-metabolic profile of infiltrating macrophages and monocyte to better evade host immunity and enhance tumor progression and metastasis [24]. Other mechanisms associated with the mobilization and polarization of macrophages by hypoxia induction include the exosomal delivery of miRNA-1246 to target TERF2IP (telomeric repeat-binding factor 2-interacting protein) via the STAT3 (signal transducer and activator of transcription 3) and NF-κB (nuclear factor kappa light chain enhancer of activated B cells) [91] and the phosphatase and tensin homolog (PTEN)/phosphatidylinositol 3-kinase γ (PI3Kγ) [78] pathways. Hypoxic exosomes obtained from pancreatic tumor cells trigger macrophages to the M2 phenotype in an HIF1a or HIF2a-dependent manner, leading to facilitated migration, invasion, and epithelial–mesenchymal transition of pancreatic cancer cells. Further analysis revealed that miR-301a-3p is enriched in the hypoxic pancreatic cancer cell-derived exosomes, the level of which positively correlates with the depth of invasion, lymph node metastasis, late TNM (tumor node metastasis) stage, and poor prognosis of pancreatic cancer. The hypoxic exosomal miR-301a-3p triggered the M2 macrophage polarization through the PTEN/PI3Kγ signaling pathway [78]. M2 macrophage-derived exosomal miR-501-3p also inhibits the tumor suppressor gene TGFBR3 and encourages the development of pancreatic ductal adenocarcinoma via the activation of the TGF-β signaling pathway [92]. Tumor cell-released autophagosomes convert macrophages into anti-inflammatory M2-like phenotypes, characterized by the expression of PD-L1 and IL-10. These macrophages also inhibit the proliferation of CD8+ and CD4+ T cells in vitro and enhance tumor growth, mainly through PD-L1 in vivo [93]. It is reported that hypoxia induces a high expression of miR-940 in exosomes derived from epithelial ovarian cancer, capable of stimulating M2 macrophage phenotype polarization to encourage cancer proliferation and migration [94].

### 5.2. Monocytes

Indirectly, monocytes influence immune evasion by differentiating into macrophages and myeloid lineage DCs and influence both innate and adaptive immunity. Directly, monocytes activated within the TME strongly express PD-L1 proteins, which are further enhanced by monocyte-secreted IL-10 and TNFα. The PD-L1(+) monocytes potently inhibit tumor-specific T cell immunity and encourage tumor growth [95]. Tumor cells constantly produce a large number of exosomes that interact with primary monocytes and induce an activated phenotype that collaborates with other microenvironment factors to form the immune-suppressive milieu of the tumor environment [96]. Monocytes efficiently take up exosomes expressed by both primary and metastatic isogenic colorectal cancer cells. The internalized vesicles reprogram the immunophenotype and secretory profile of both monocytes and inactive macrophages, triggering a mixed M1 and M2 cytokine response [97]. Tumor cell-released autophagosomes polarize monocytes to an M2-like phenotype that possesses an elevated expression of PD-L1, IL-10, and CD163, but reduced expression of the MHC II cell surface receptor HLA-DR (human leukocyte antigen—DR) with T cell-suppressive functions. In addition, the concentration of LC3B+ extracellular vesicles appeared to markedly correlate with the increased expression of PD-L1 and IL-10 in monocytes from ascites or effusions of cancer patients [93]. Another study found that in hypoxic conditions, endometrial cancer KEL cells enhance monocyte THP-1 cell transformation to M2-like macrophage phenotype via the delivering of exosomal miRNA-21, serving as a potential mechanism for the formation of the tumor-friendly microenvironment that favors endometrial cancer progression. Notably, there were enhanced mRNA expression levels of IL-10 and CD206 in monocyte cells [98]. Hypoxia promotes tumor secretion of biomolecule-loaded exosomes that can modify the immunometabolic profile of infiltrating monocyte–macrophages to better evade host immunity and enhance tumor progression. [24].

### 5.3. Natural Killer (NK) Cells

NK cells are dysregulated within the TME, rendering them ineffective in eradicating the cancer cells. This is due to immune-suppressive factors, such as hypoxic exosomes from the tumor and stromal cells, Tregs, and varying kinds of soluble factors, including growth factors, reactive oxygen species, and cytokines [99]. Natural killer group 2, member D (NKG2D) is an activating cell surface receptor that is abundantly and predominantly expressed on cytotoxic immune cells, such as NK cells, NKT cells, CD8+ T cells, and subsets of γδ T cells. Hypoxic cancer cell-derived microvesicles transfer TGF-β1 to NK cells that reduce cell surface expression of the activating receptor NKG2D, resulting in inhibition of NK cells’ function and their antitumor response. Additionally, miR-23a in hypoxic tumor-derived microvesicles functions as an extra immune evasive factor by directly targeting the expression of CD107a in NK cells [100]. Although the interaction between NK and the myeloid cell can elicit antitumor activities, TME-derived myeloid cells, including DCs, macrophages, neutrophils, and MDSCs, often dysregulate NK cells through direct cell-to-cell interactions, downregulation of key NK cell receptors, depletion of growth factors and nutrients necessary for NK cell growth, and expression of chemokines, metabolites, and cytokines that ultimately change NK cell cytotoxicity, trafficking, and survival [99,101,102]. Tumor-associated macrophages and MDSCs are usually the main myeloid cell populations in aggravated TME and constitute the key producers of suppressive TGF-β and IL-10 that promote NK cell dysfunction [103].

### 5.4. T Cells

A T cell is a type of lymphocyte and a crucial leukocyte that plays a central role in the adaptive immune response. In demonstrating the effects of hypoxia on mitogen-stimulated T cells, the authors noticed that oxygen depletion reduces T cell activation via the HLA-DR expression, inhibits T cell proliferation, and reduces pro-inflammatory, but upregulates anti-inflammatory, cytokine secretion. Mechanistically, there was increased expression of PD-1, FOXP3 (forkhead box P3), and TGFβ1, key players in the tumor immune evasion response, while genes involved in the inflammatory response, such as IL2 and interferon (IFN)γ, were downregulated [104]. Cytotoxic T-lymphocyte-associated protein 4 (CTLA-4), also known as CD152, is a protein receptor that functions as an immune checkpoint and downregulates immune responses. Hypoxia is associated with tumor resistance to T cell infiltration even in the context of CTLA-4 and PD-1 blockade. The reduction in or elimination of hypoxia in tumors drives an influx of T cells into hypoxic zones, contributes to reduced MDSCs density, and effectively decreases the ability of the tumor to replenish [105]. The accumulation of CD39+CD8+ T cells indicates poor cancer prognosis due to decreased antitumor immune response, including downregulated expression of TNF-α and IFN-γ, with elevated PD-1 and TIM-3. There is also a positive correlation between increased infiltration of CD39+CD8+ T cells and Tregs and M2-macrophage polarization in renal cell carcinoma patients [106]. Serum exosomal miR-24-3p level is reported to correlate with worse disease-free survival in patients with nasopharyngeal carcinoma and impedes T-cell proliferation and Th1 and Th17 differentiation, but induces Treg differentiation of lenti-shFGF11 (fibroblast growth factor 11)-transfected T cells. Further analysis indicated that hypoxia upregulates cellular and exosomal miR-24-3p levels and enhances the inhibitory effect on T-cell proliferation and differentiation by repression of FGF11 [57].

### 5.5. γδ. T lymphocytes

**Figure 4:**
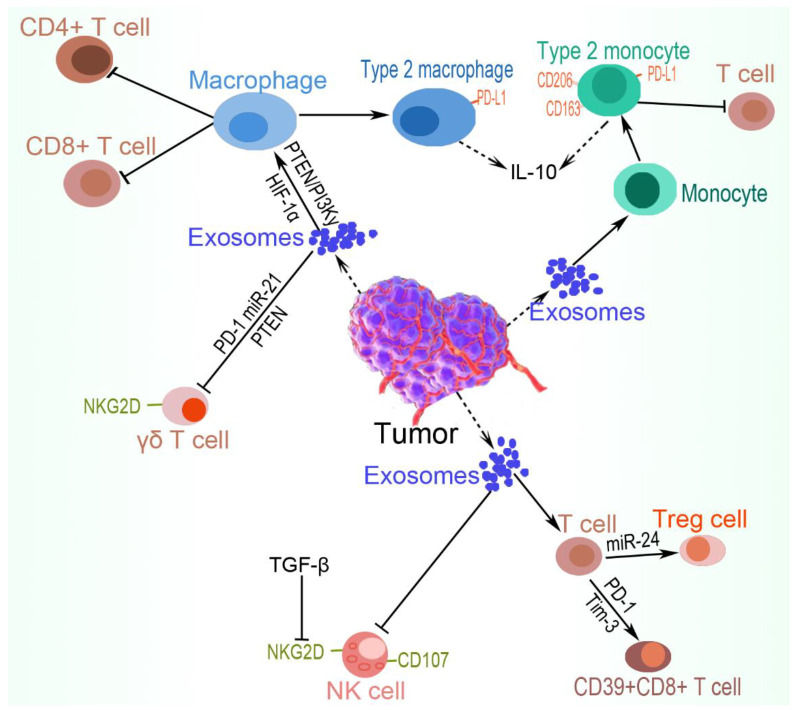
Tumor cell immune evasion via the suppression of immune cells. Tumor cells avoid immune surveillance by deactivating antigen-presenting cells, such as NK cells, via inhibiting their surface molecules, such as NKG2D and CD107, and polarizing macrophages and monocytes into tumor-promoting M2 phenotype. Cytotoxic T cells are deactivated or induced to differentiate into Tregs, in addition to expressing certain tumor immune evasion surface recognition molecules. Moreover, the unique T lymphocyte subtype γδ T cells are deactivated via the inhibition of surface molecules, such as NKG2D and exosomal transfer of PD-1, PTEN, and miR-21. The resultant tumor-friendly phenotypes participate in further suppressing the immune systems to assist tumor immune evasion.

Tumor cells escape immunosurveillance through various mechanisms and tumor cell metabolism, including the high oxygen demand that affects the metabolic states and functions of tumor-infiltrating lymphocytes, such as γδ T cells [107]. As a unique lymphocyte population, γδ T cells have been reported to possess either anti- or pro-tumoral functions in several cancer types. In the TME, tumor-derived exosomes alter the cytotoxicity and expansion of γδ T cells in a 70 kilodalton heat shock protein (Hsp70s or DnaK)-dependent, but DC-independent, manner. Moreover, hypoxic tumor-derived exosomes enhance the suppressive effect of myeloid-derived suppressor cells (MDSCs) on γδ T cells via the miR-21/PTEN/PD-L1 regulatory axis [108]. Contrary to this observation, Liu and colleagues report that the ability of tumor-derived exosomes to stimulate T cells is typically dependent on the recognition and presentation of antigens by DCs [109]. The differences could be attributed to the fact that γδ T cells have characteristics of T cells, NK cells, and antigen-presenting cells [110]. Pro-tumoral functions of IL-17-producing γδ T (γδ17T) cells have also been documented, where the γδ17T cells produced IL-8, TNF, and granulocyte–macrophage colony-stimulating factors to induce the accumulation of anti-inflammatory MDSCs in the TME and drive pro-tumoral inflammation [111]. Again, γδ T cells have been suggested to assist pancreatic oncogenesis via restraining αβ T-cell activation in the TME [112]. Tumor hypoxia induces the γδ T cell PKA (protein kinase A) pathway at the transcriptional level, causing the inhibition of the activator receptor NKG2D [113]. Hypoxia-exposed γδT cells exhibit decreased cytotoxicity against oral tumor cells due to hypoxia-induced reduction in calcium efflux and the expression of degranulation marker CD107a in γδT cells [114]. The hypoxic tumor-derived exosomal regulation of macrophages, monocyte, NK cells, and T cell types to facilitate immune evasion is summarized in Figure 4.

### 5.6. Myeloid-Derived Suppressor Cells (MDSCs)

MDSCs are a heterogeneous population of immature myeloid cells that suppress innate and adaptive immunity [115]. MDSCs originate from the bone marrow stem cells and strongly expand in pathological conditions, such as chronic infections and cancer, as a result of altered hematopoiesis [116,117]. In the TME, MDSCs constitute a key tumor promoting factor, which is further regulated by hypoxia-induced exosomes taken up by MDSCs. The hypoxia-inducible expression of exosomal RNAs, such as miR-10a and miR-21, in glioma cells mediate the induction of MDSC expansion and activation via targeting RAR-related orphan receptor alpha (RORA) and PTEN [56]. In a similar study, the exosomal miR-29a and miR-92a activated the proliferation and function of MDSCs by targeting the high-mobility group box transcription factor 1 (Hbp1) and protein kinase cAMP-dependent type I regulatory subunit α (Prkar1α), respectively [118]. Granulocytic MDSCs express high concentrations of exosomal S100A9 that enhance colorectal cancer cell stemness and the susceptibility of mice to azoxymethane/dextran sulfate sodium-induced colitis-associated colon cancer. Further analysis indicates that hypoxia induces granulocytic MDSCs to produce more S100A9-enriched exosomes in an HIF-1α-dependent manner, leading to promoted colorectal cancer cell stemness and growth [119]. Other mechanisms by which MDSCs mediate immune suppression within the tumor and inflammatory microenvironment include exosomal S100A9 via the RAGE and toll-like receptor (TLR)4 signaling pathways [120], the interferon regulatory factor 7 (IRF7) [121], and exosomal prostaglandin E2 by enhancing IL-10+ B Cells [122].

### 5.7. Dendritic Cells

DCs are the most active antigen-presenting cells and relate to the innate and adaptive immune responses [123]; hence, for a tumor cell to survive and progress, it must evade DCs. DCs collect antigens, process them, and present typical antigenic structures to lymphocytes [124]. They travel through lymphatic vessels to present these antigens to T cells in the lymph nodes under the directional influence of the C-C chemokine receptor type 7 (CCR7) [125]. Hypoxia induces the upregulation of miRNA 21 in DCs to participate in the suppression of CD80, CD86, and MHCII (major histocompatibility complex class II molecules) on DC surfaces, leading to decreased secretion of inflammatory cytokines and CCR7 [126]. Decreased expression of CD80 and CD86 compromises DC differentiation and maturation [127], while decreased surface MHCII impedes the antigen-presentation ability of DCs and the production of IL-12, capable of inducing the differentiation and proliferation of helper T (Th)1/Th2 cells [128,129]. Certain antitumor therapies, such as sorafenib, are known to promote immune evasion by increasing hypoxia in the TME, coupled with an upregulated expression of inhibitor PD-L1 and Tregs in tumor tissues. However, in the treatment of DCs with exosomes derived from orthotopic hepatocellular carcinoma, model cells enhanced the number of CD8+T cells but decreased Tregs, with a significantly elevated number of PD-1+CD8+T cells [130]. In an inflammatory environment, HIF-1α limits plasmacytoid DC (pDC) generation in the bone marrow, while the absence of a functional HIF-1α in myeloid cells markedly enhances the numbers of pDC within tumors as compared to wild-type controls [131]. Moreover, the deficiency in HIF-1α in DCs inhibits their ability to induce Treg proliferation in secondary lymphoid organs and disrupts Treg homing towards inflamed guts [132].

### 5.8. B Cells

As a common feature of human solid tumors, hypoxia plays a multifaceted role in cancer progression. Tumor cell-released autophagosomes are sufficient to suppress the antitumor immune response in the mouse by inducing IL-10-producing B cells through high-mobility group B1 (HMGB1) [133]. A similar study found that hypoxia or starvation exerts an immune evasion effect by upregulating HMGB1 on tumor cell-released autophagosomes. Moreover, hypoxic tumor cell-released autophagosomes from human hepatocellular carcinoma cell line HepG2 induce more IL-10-producing B cells, with suppressive functions on CD4+ and CD8+ T cells [134]. Regulatory B cells are a significant feature of the glioblastoma microenvironment in both clinical and preclinical model samples. In tumors, regulatory B cells are characterized by immune-suppressive functions toward activated CD8+ T cells, the production of anti-inflammatory cytokines TGFβ and IL10, and the overexpression of inhibitory molecules PD-L1 and CD155, leading to glioblastoma progression. Mechanistically, MDSCs promote regulatory B-cell function via microvesicle delivery of membrane-bound PD-L1 to the tumor-infiltrating B cells [135]. Tumor cell-released autophagosomes have also been demonstrated to decrease antitumor immunity by promoting anti-inflammation through the induction of M2-like macrophages with increased expression of PD-L1 [93], inducing CD4+ T cell-mediated immune suppression via a TLR2-IL-6 cascade [136] and triggering IL-10-producing B cells, with suppressive activity on T lymphocytes via the TLR2-MyD88-NF-κB signal pathway [133]. Autophagosome-derived exosomes obtained from malignant effusions of cancer patients could activate the differentiation of human B cells into IL-10-producing B cells with immunomodulatory activities, with the level positively correlating with HMGB1 expression on tumor cell-released autophagosomes [133].

### 5.9. Mast Cells

**Figure 5:**
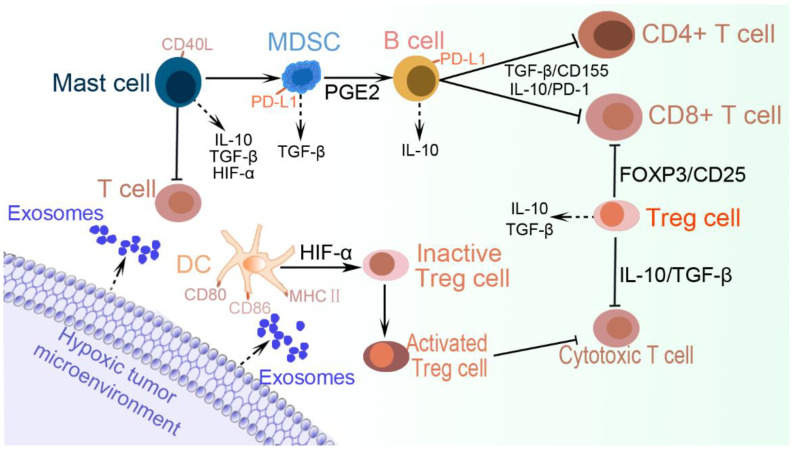
Tumor immune evasion via increased recruitment of tumor-promoting immune cells. The hypoxic TME expresses exosomes that upregulate the activation of anti-inflammatory cells including MDSCs and Tregs, and their associated cytokines, such as IL10, TGF-β, and HIF-1α, among other surface recognition molecules that together facilitate tumor cell immune evasion. Certain phenotypes of DCs and mast cells increase the activation of Tregs and MDSCs, respectively. MDSCs further induce B cells via PGE2 to secret IL10 and deactivate both CD8+ and CD4+ T cells, while Tregs and mast cells also inhibit cytotoxic T cells. The increased recruitment, activation, and expansion of these tumor-friendly cells provide the conducive microenvironment needed for immune evasion and tumor progression.

Mast cells can promote adenocarcinoma development by orchestrating tumor immune-evasion activities. Mast cells expressing CD40L restore polymorphonuclear myeloid-derived suppressor cell (PMN-MDSCs) functions, induce T-cell unresponsiveness, and encourage adenocarcinoma development [137]. HIF-2α triggers the expression of stem cell factor (SCF), which serves as a chemoattractant for mast cells. Tumor-infiltrating mast cells impair antitumor immunity, partly by secreting IL-10 and TGF-β. This also impairs the function of CD8+ T cells [138]. Mast cell-derived HIF-1α in the TME is associated with further expressions of HIF-1α and VEGF in mast cells that enhance tumor growth. They also secrete histamine that induces new vessel formation by acting through the histamine1 (H1) receptors [139]. It has also been demonstrated that mast cells are located preferentially in hypoxic zones of melanoma tumors, where hypoxia-induced CCL-2 production in mast cells requires an increase in calcium, mediated by glutathionylation and membrane translocation of L-type voltage-dependent Ca2+ channels [140]. Moreover, CCL5-dependent mast cell infiltration facilitates suppression of antitumor activities within the TME, resulting in tumor progression and adverse survival outcomes in clear cell renal cell carcinoma patients. This is also associated with increased expression of genes encoding IFN-α, IFN-γ, IL-6, TNF-α, JAK (Janus kinase)-STAT3, and NF-ΚB [141]. Mast cells contain proangiogenic factors, in particular tryptase, associated with increased angiogenesis in several tumors. To that effect, a recent study concluded that mast cells carrying c-Kit receptors and tryptase are strongly associated with angiogenesis in pancreatic ductal adenocarcinoma tissue [142]. However, the genetic ablation of mast cells in the transgenic adenocarcinoma mouse prostate (TRAMP) model restores their capability to induce a tumor-specific cytotoxic T-cell response, which correlates (in Kit^Wsh^-TRAMP mice) with decreased activity of PMN-MDSCs and downregulated expression of Arg1, Stat3, and Nos2 [137]. Figure 5 presents a tumor-promoting microenvironment as exerted by hypoxic exosomes via the regulation of MDSCs, mast cells, B cells, DCs, and Tregs.

### 5.10. Regulatory T Cells

Tregs and tumor–exosomes are key mediators in preventing the rejection of malignant cells. Tumor-derived exosomes and CCL20 facilitate Treg recruitment as well as conventional CD4(+)CD25(−) T cells, which are converted into inhibitory CD4(+) CD25 (high) cells. Moreover, the tumor-derived exosomes induced Treg expansion, resulting in the generation of Tim3 (Low) Tregs with increased expression of CD25 and FOXP3. These findings are consistent with a stronger suppression of responder immune cell proliferation and the expression of anti-inflammatory cytokines, including IL10 and TGF-β1 [143]. The upregulated expression of IL-23 activates Treg proliferation and promotes IL-10 and TGF-β expression, resulting in the suppression of tumor cell killing by cytotoxic lymphocytes [144]. In nasopharyngeal carcinoma, the EBV-EBNA1 molecular mechanism is identified as a potent stimulator of chemotactic migration of Tregs toward the TME via upregulation of the TGF-β1-SMAD3 (mothers against decapentaplegic homolog 3)-PI3K-AKT-c-JUN-CXCL12 (C-X-C motif chemokine 12)-CXCR4 (C-X-C chemokine receptor type 4) axis and downregulation of miR-200a. Moreover, EBV-EBNA1 induces the chemoattraction of Tregs by regulating the protein–protein interactions of the SMAD3/c-JUN complex in a TGFβ1-dependent manner in vitro and in vivo [145]. A representative overview of immune cells and associated mechanisms, as related to hypoxic exosomal regulation in the tumor, is presented in Table 2.

## 6. Escape of Immunogenic Cell Death via DAMPs

Damage-associated molecular pattern (DAMP) molecules are proteins produced by cells subjected to stressful conditions, such as hypoxia, leading to the activation of innate immunity and providing a pathway to a systemic inflammatory response in the absence of infection. DAMPs promote cancer growth via the regulation of inflammation in the TME, enhancing angiogenesis and increasing autophagy with the evasion of apoptosis [148,149]. DAMPs, such as nuclear high-mobility group box 1 (HMGB1) and mitochondrial DNA (mtDNA), are released by hypoxia-induced intracellular translocation and couple together to form a complex that stimulates TLR9 signaling pathways to facilitate tumor cell proliferation [150]. Several exosomal miRNAs, such as lncRNA-TP73- AS1 [151], miR-325 [152], miRNA-129-5p [153], miR-200a, miR-21, miR-325, miR-320a, and miR-505 [154,155], in tumors have been identified as modulators of HMGB1-mediated biological actions within the TME, including immune evasion, cell migration, and tumor metastasis, hence, facilitating cancer development [156]. HMGB1 is also associated with resistance of tumor cells to immunotherapy [157], chemotherapy [158], and suppression of antitumor immunity via the induction of Tregs within the TME [159].

## 7. Escape from Immune Surveillance via Surface Recognition Molecules

Hypoxic-tumor-derived exosomes contribute to elevated expression of certain cell surface molecules on both tumor and immune cells, among others. These surface proteins increase tumor immune evasion activities and help tumor cells avoid surveillance.

### 7.1. PD-1/PD-L1

PD-1/PD-L1 is an important immune checkpoint and the dysfunction of this axis significantly contributes to immune escape and tumor metastasis. Transcriptional factors, such as HIF, PTEN, CDK5, p53, BRD4, and STAT, alongside signal networks, including PI3K/Akt/mTOR and MAPK, induce and modulate PD-1/PD-L1 expression [160,161]. Recent investigations have explored the crucial role of PD-L1 as a transmembrane protein in exosomes and found exosomal PD-L1 as a mechanism of tumor immune escape and immunotherapy resistance. Exosomal PD-L1 triggers systemic suppression of antitumor functions, represses T cell effector function, and transfers functional PD-L1 across the TME [162]. Tumor-derived exosomal PD-L1 inhibits T cell activation in the draining lymph node and rescues the growth of tumors unable to secrete their own PD-L1. Conversely, the removal of exosomal PD-L1 suppresses tumor growth, even in models resistant to anti-PD-L1 antibodies [163]. This immune checkpoint is expressed on several immune cell types, including MDSCs [164], tumor-associated macrophages [165], B cells [166], tumor-infiltrating lymphocytes [167], and MDSC-induced PD-1 -PD-L1+B-cell subset [168], among others. As the detrimental effects of hypoxia within tumors are associated with the induction of a tumor-friendly microenvironment, there is an interaction between tumor hypoxia dynamics and the PD-1/PD-L1 axis. Head-and-neck squamous cell carcinoma patients with persistent tumor-associated hypoxia during therapy and PD-L1 expression on tumor cells exhibit a worse outcome [169].

### 7.2. CD73/CD39

The CD39/CD73–adenosine pathway has been demonstrated as a crucial tumor-induced immune-suppressive mechanism [170]. A study reported that CD73 and CD39 are not expressed by normal alveolar and bronchial epithelium but overexpressed by cancer cells, tumor-infiltrating lymphocytes, and cancer-associated fibroblasts. While elevated expression of CD73 in cancer cells is directly associated with HIF1α and lactate dehydrogenase 5 (LDH5) expressed by cancer cells, the expression of CD39 by cancer-associated fibroblasts is directly linked with PD-L1 expression by cancer cells. Moreover, a high stroma expression of CD39 and CD73 results in significantly abundant FOXP3+ and PD-1+ tumor-infiltrating lymphocytes in tumors and hypoxia and acidity induce CD73 mRNA and protein levels in cancer cells [171]. CD39 and CD73 enhance immune-evasive activities of tumor and stromal cells by impeding cytotoxic T cell function and NK cell cytotoxicity, but increasing Treg function via the degradation of immune stimulatory ATP to adenosine. The resultant outcome is associated with increased mortality in ovarian cancer patients [172]. The co-incubation of CD4(+) CD39(+) Tregs with B cells, CD4(+) CD73(+) T cells, or CD39(+) CD73(+) exosomes produce immune evasive adenosine. This implies that contact with membrane-tethered CD73 is sufficient for adenosine production by CD4(+) CD39(+) Tregs. The authors further demonstrated that in the microenvironments containing CD4(+) CD73(+) T cells, B cells, or CD39(+) CD73(+) exosomes, CD73 is readily available to CD4(+) CD39(+) CD73(neg) Tregs for the production of tumor-promoting adenosine [173]. CD39 and CD73 are also associated with CD19+ extracellular vesicle production from B cells that impair CD8+ T cell responses. Increased hypoxia enhances the secretion of CD19+ exosomes that triggers Rab27a mRNA transcription, which negatively affects tumor chemotherapy [174].

### 7.3. CD38

CD38, also known as cyclic ADP ribose hydrolase, is a glycoprotein located on the surface of some immune cells, such as CD4+ T cells, CD8+ T cells, NK cells, and B lymphocytes, as well functions in signal transduction, cell adhesion, and calcium signaling [175]. CD38 promotes tumor cell proliferation, inhibits cell senescence, and enhances cell metastasis by regulating the metabolic-associated signaling pathways linked with tumor protein 53, HIF-1α, and sirtuin 1 [176]. CD38 is expressed on the surface of secreted exosomes derived from immune cells, such as lymphoblastoid B cells, where the exosomal CD38 is associated with signaling molecules CD81, Hsc-70, and Lyn. CD38 is enzymatically active in both exosomes and membrane rafts and could ligate to induce Akt/PKB and Erk activation [177]. As an alternative immune checkpoint molecule that is involved in tumor immune-evasive responses, CD38 is associated with resistance to immune checkpoint inhibitor therapy and likely participates in hyper-progressive tumor growth via the activation of hypoxic pathways, adenosine receptors, and activation-induced cell death (AICD)-dependent T-cell depletion [178]. In the hypoxic TME, the increased enzymatic functions of CD38 result in an tumor-friendly environment, leading to increased immune cell resistance in tumors as well as faster growth and proliferation rates [179].

### 7.4. CD47

CD47, also known as integrin-associated protein, belongs to the immunoglobulin superfamily. It acts as a ‘don’t eat me’ signal to macrophages and recruits the inhibitory immunoreceptor signal regulatory protein α (SIRPα) [180]. CD47 serves as a tumor-associated cell surface recognition protein and is associated with chemoresistance [181], while tumor hypoxia and HIF-1 are involved in indirectly activating the translation of CD47 to induce the mechanism of immune evasion in tumors [62]. A reduction in the CD47/SIRPα signaling pathway via PD-1 blockade significantly reduced tumor growth by downregulating tumor immune-suppressive network mediators, such as MDSCs, tumor-associated macrophages, DCs, as well as effector T cells [182]. Zhang and colleagues report that the deficiency in CD47 leads to cancer stem cell depletion and datasets derived from thousands of breast cancer patients revealed that CD47 expression correlates with HIF target gene expression and with patient mortality [62]. The elevated expression of CD47 in cancers prevents macrophage phagocytosis and the anti-human CD47 antibody B6H12 prevents tumor growth in several xenograft models by preventing SIRPα engagement. Mechanistically, B6H12 treatment facilitates the expression of exosomal microRNA-7 in breast cancer stem cells, which targets EGFR and KLF4 mRNAs, leading to decreased EGFR, KLF4, and EGF-induced EGFR tyrosine phosphorylation [183].

### 7.5. Other Surface Recognition Molecules

Cancers protect themselves from immune attacks by stimulating immune checkpoint targets to elicit immune evasion and tumor tolerance. In addition to PD-1, also known as CD279, other inhibitory immune checkpoints known to induce a negative signal to T cells include cytotoxic T-lymphocyte antigen 4 (CTLA-4), also known as CD152, lymphocyte-activation gene 3 (LAG-3), V-domain immunoglobulin suppressor of T cell activation (VISTA), and T cell immunoglobulin and mucin domain 3 (TIM-3). Patients with cancers, such as human hepatocellular carcinoma, exhibit significantly higher T cell immunoglobulin domain and mucin domain-1 (TIM-1)^+^Breg cell infiltration in their tumor tissue compared with paired peritumoral tissues. The infiltrating TIM-1+Breg cells show a CD5^high^CD24^−^CD27^−/+^CD38^+/high^ phenotype, express high quantities of the anti-inflammatory cytokine IL-10, and exhibit strong inhibitory function against CD8+ T cells. Exosome-derived HMGB1 stimulates B cells and enhances TIM-1+Breg cell expansion via the TLR 2/4 and mitogen-activated protein kinase (MAPK) signaling pathways [184]. T cell immunoglobulin and ITIM domain (TIGIT) is a checkpoint receptor identified to participate in the mediation of T cell exhaustion and dysfunction of NK cells in tumors. A study found that TIGIT, but not the other checkpoint molecules PD-1 and CTLA-4, was linked with NK cell exhaustion in both humans and tumor-bearing mice with colon cancer [185]. The human CD94/NKG2A is an inhibitory receptor that recognizes HLA-E and is expressed by NK cells and a subset of T cells [186]. CD94/NKG2A decreases antitumor immunity by deactivating the cytotoxicity in both NK cells and CD8+ T cells on tumor cells in mice and humans [187].

## 8. Escape from Immune Surveillance via Antitumor-Suppressive Molecules

**Figure 6:**
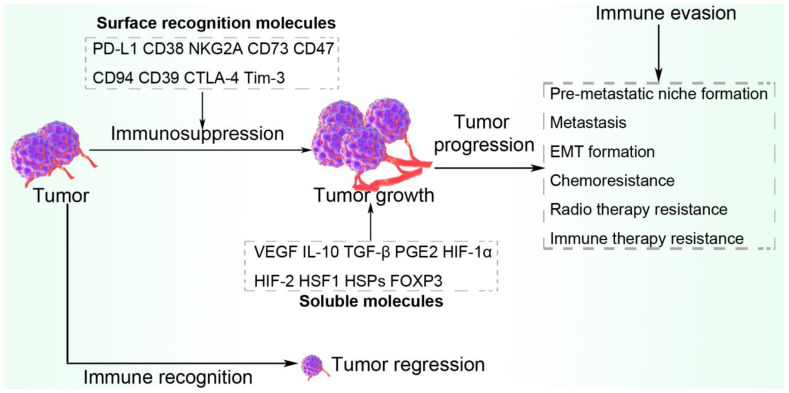
Hypoxic exosome-induced surface recognition molecules and soluble factors. Exosomes produced in the hypoxic TME enhance the expression of both surface recognition molecules and soluble factors that together promote tumor immune evasion. The exosomes either carry these molecules as packages or trigger their production via their regulatory activities. Tumors that undergo immune recognition gradually decrease in size while immune evasion causes increased tumor size and progression.

Tumor hypoxic stress causes HIF-1α to mediate the secretion of a variety of immune evasion-promoting molecules from tumor cells, including TGF-β, VEGF, IL-10, and PGE2. These tumor-enhancing molecules assist the tumor cell to escape immune system surveillance and attack. For example, HIF-1 couples with the COX-2 promoter at the HRE3 site, upregulating the expression of COX-2 and promoting the production of PGE2. Secreted PGE2 participates in tumor proliferation and invasiveness. Under hypoxic conditions in the tumor, PGE2 is upregulated through the HIF-1/COX-2 pathway, suppressing the immune system and enhancing tumor cell escape from immune surveillance by activating PD-L1 expression and suppressing the maturation of DCs [22,188]. The combined effect of tumor-promoting soluble factors and surface recognition molecules within the hypoxic TME is summarized in Figure 6.

### 8.1. TGF-β

TGF-β suppresses type 2 immunity to cancer and serves as an important regulatory axis for tumor progression [189]. Several therapies have explored the inhibition of this tumor-promoting molecule as a means of fine-tuning the TME to enhance tumor cell death [190,191]. Hypoxia in solid tumors induces increased expression of TGF-β1 and HIF-1α, which facilitates tumor growth by inhibiting the antitumor immune response and inducing tumor immune escape [192]. The depletion of TGF-β-R2 in CD4+ T cells impedes tumor progression as a result of tissue healing and remodeling of the blood vasculature, resulting in tumor cell hypoxia and death in distant avascular regions. This host-directed antitumor protective response is also dependent on the Th2 cytokine IL-4 [193]. Tumor-derived exosomes contain pro-EMT molecules, such as TGFβ, HIF1α, and β-catenin, that do not only aid tumor cell immune evasion but enhance the invasive and migratory abilities of recipient cells, hence, contributing to stromal remodeling and premetastatic niche formation [194]. Tumor-derived TGF-β stimulates CD39 and CD73 expression, thereby inhibiting NK cell and T cell functions and protecting tumor cells from the cytotoxic effect of chemotherapy via ectonucleotidase activity, where TGF-β triggers the phosphorylation of mTOR and the subsequent activation of HIF-1α to facilitate the expression of CD39/CD73 on MDSCs. The resultant immune-suppressive and chemo-protective effects encourage tumor cell immune evasion and cancer progression [170].

### 8.2. IL-10

IL10, also known as human cytokine synthesis inhibitory factor (CSIF), is a key anti-inflammatory cytokine that inhibits pro-inflammatory responses of both innate and adaptive immune cells. Tumor-derived exosomes modulate immune cells within the TME to secrete large quantities of IL-10 to assist tumor cells to evade immune surveillance. Hypoxia-induced-HIF-2α contributes to evasion of antitumor immunity via SCF secretion and subsequent recruitment of mast cells, which impair antitumor immunity, partly by secreting IL-10 and TGF-β [138]. Hypoxia tumor-derived exosomal-hsa-circ-0048117 is transmitted to macrophages to promote their polarization to M2 macrophages, which enhance the invasive and migratory ability of tumor cells through secreting IL-10, Arg1, and TGF-β [195]. Under hypoxia, MDSCs are enhanced to express IL-10 and IL-6 to promote the suppressive activity of MDSCs [196]. In the clear cell renal cell carcinoma microenvironment, glutamine consumption by the tumor cells causes the local depletion of extracellular glutamine, resulting in IL-23 secretion by tumor-infiltrating macrophages via the stimulation of HIF1α. Consequently, IL-23 induces Treg proliferation and promotes IL-10 and TGF-β expression, hence, suppressing tumor cell killing by cytotoxic lymphocytes [144]. Moreover, intermittent hypoxia in a solid tumor environment induced the upregulation of tumor-evasive factors, such as IL-10, HIF-1α, TGF-β1, and TNF-α [192].

### 8.3. PGE2

PGE2 has been demonstrated to induce HIF-1α activation in a relatively less hypoxic microenvironment during the early stages of the tumor. The authors found that PGE2 mediates early HIF-1α stimulation and subsequent hypoxia-induced HIF-1α stimulation that further facilitates PGE2 synthesis, driving an antitumor suppressive response via the recruitment and functional alteration in tumor site macrophages with the ability to reduce pro-inflammatory gene expression and induce T cell suppression [197]. Under hypoxic conditions, the PGE2/COX-2 axis effectively stabilized HIF2α and its activity, enhancing the activation of VEGF, cyclin D1, and the TGFα/EGFR pathway to mediate hepatocellular carcinoma development and reduce the sensitivity to the antineoplastic therapy, sorafenib [198]. Exosomes from pancreatic ductal adenocarcinoma cells induce elevated production of PGE2 from macrophages and activate their polarization to the anti-inflammatory M2-like phenotype [199].

### 8.4. VEGF

As the most potent cytokine involved in tumor angiogenesis and metastasis formation, VEGF is induced by hypoxia to promote antitumor therapy resistance via the HIF2-VEGF axis [200], in addition to endothelial cell proliferation, migration, and survival [201]. The inhibition of VEGF is associated with increased antitumor immune responses. In one such study, blockade of VEGF-A with a mAb to VEGF augmented the activation of CD8+ T cells within tumors and potentiated their capacity to produce cytokines (IFNγ, TNFα). Further analysis indicated reduced expression of the inhibitory receptors PD-1 and T cell immunoglobulin and mucin domain-containing protein 3 (TIM3), but increased tumor antigen-specific CD8+ T cells and CD8+ T cell receptor OX40, a member of the tumor necrosis factor receptor superfamily that function in antigen presentation [202].

### 8.5. Others

HSPs have been identified in exosomes, membrane surfaces, oncosomes, as well as free HSPs in tumors. The HIF-1 and heat shock factor 1 (HSF1) contribute to the induction of the expression of HSPs, while matrix metalloproteinase 3 and heterochromatin protein 1 are novel inducers of HSPs. Oncosomes released by tumor cells and transfer of HSPs is a major aspect of RASP, by which immune evasion can be established, facilitating tumor progression and resistance against stressful microenvironment factors, such as hypoxia, radiation, drugs, and immune systems [74,203]. In effect, exosomal HSP/CD91 signaling in cancer cells promotes cancer progression [74]. CCL20 allows the intratumoral recruitment of human Tregs in nasopharyngeal carcinoma. Exosomes derived from the tumor caused the expansion of Tregs, inducing the generation of Tim3(Low) Treg with increased expression of CD25 and FOXP3 [143]. Hypoxia-inducible protein 2 (HIG2, also known as HILPDA) is highly expressed in tumors, including gliomas, and correlates with tumor grade and poor patient prognosis. In the hypoxic TME, HIF1α binds to the HIG2 promoter and increases the angiogenesis gene VEGFA, and this may encourage tumor resistance to anti-angiogenesis treatments [204].

## 9. Conclusions and Perspective

The metabolic features of the TME are characterized by a critical level of hypoxia, extracellular acidosis, significantly raised concentrations of adenosine, elevated lactate levels, and depleted nutrient resources. These tumor microenvironmental features are major drivers of genetic instability, malignant progression, intratumor heterogeneity, and the development of resistance to conventional anticancer therapies. Hypoxia-dependent HIF-1α activation serves as an important factor in the orchestration of a multifaceted suppression of adaptive and innate antitumor immune responses, as well as immune-based tumor treatments. These characteristic features induce strong antitumor-suppressive signals, including the stimulation, recruitment, and expression of anti-inflammatory cells, such as Tregs, MDSCs, and TH2-type cytokines, and the polarization of antigen-presenting cells, such as macrophages, monocytes, and DCs into tumor-friendly phenotypes. Moreover, there is inhibition of antitumor immune responses orchestrated by DCs, CD4+ T cells, NK cells, NKT, and CD8+ T cells, and impeded expression of immune-stimulatory Th1-type cytokines.

Hypoxia, being a crucial feature in tumors, participates in remodeling the TME to assist tumor progression. In so doing, it triggers the release of a high quantity of tumor-derived exosomes, which extensively communicate with their microenvironment to concoct conditions favorable for tumor cell survival, growth, and metastatic spread. The expressed cancer-derived exosomes contain elevated levels of exosomal cargos derived from the hypoxic microenvironment, which induce immune suppression, escape of immune surveillance, and encourage the malignant properties of cancer cells. Hypoxic tumor-derived exosome-induced immune evasion includes the inactivation of cytotoxic cells, polarization of antigen-presenting cells into anti-inflammatory phenotypes, expression of immune evasion-promoting soluble factor and surface receptors, and increased recruitment of anti-inflammatory cells. For example, under a steady-state condition, immature myeloid cells (designated as MDSCs) differentiate into macrophages, granulocytes, and DCs. However, this differentiation is impaired in hypoxic tumors and chronic inflammatory conditions, which encourages tumor progression, resulting in the accumulation of MDSCs. These cells are capable of activating strong tumor immune-evasive effects via the expression of a variety of cytokines and immunoregulatory particles, stimulation of other tumor-promoting immune cells, inhibition of lymphocyte homing, inactivation of T cells by depleting metabolites crucial for their functions, and the production of reactive species, among others.

While hypoxia within the TME is generally associated with negative tumor prognostic outcomes, other studies have demonstrated that oxygen-depleted TMEs generate the inflammatory setting conducive to immunogenic cell death. This finding, while not extensively studied and expounded in the literature, highlights the complex nature of hypoxia-induced changes in the TME and the significance of addressing the opposing pro- and antitumorigenic features of hypoxia-driven pathways. Considering the critical interaction between hypoxia, exosomes, and the TME, future immunotherapeutic treatment modality for cancer may use a combined approach of blocking immune checkpoints, such as PD-1/PD-L1, CTLA-4 (also known as CD152), TIM-3, and LAG-3 signaling, as well as targeting HIF-1α, which may help in reversing hypoxia-induced tumor immune evasion. The complex nature of the TME also demands that efficient therapies target not only tumor-derived exosomes and hypoxia, but also neovascularization, chronic inflammation, tumor-associated fibroblast, immune cells, and the extracellular matrix. Thus, having explored the contribution of exosomal cargos, such as RNAs and proteins, as indispensable players in the cross-talk within the hypoxic TME, there are increases in the potential of targeting antitumor immunity or subverting immune evasion and enhancing tumor therapies. A single engineered nanomaterial (e.g., exosome or other nanoparticles) could be functionalized with different moieties to target different cell populations in the TME, enabling a combined strategy for cancer therapeutics. Moreover, to address challenges, such as tumor resistance or non-response to therapy, and aim at achieving personalized medicine in oncology, each tumor must be considered as a multifactorial disease, different in each patient, and, thus, require a different strategy regarding therapeutics, particularly focusing on combination therapy.

## Figures and Tables

**Table 1:** Hypoxia-induced exosomal cargos and effects on tumors.

Exosomal Component	Tumor Type/Model	Effects	Reference
Exosomal RNAs			
lncRNA-UCA1	Bladder cancer	Promotes tumor growth and progression through EMT in vitro and in vivo lncRNA-UCA1 in the human serum serves as a possible biomarker	[18]
circ-133	Colorectal cancer	Promotes cancer metastasis by acting on the miR-133a/GEF-H1/RhoA axis	[36]
miR-128-3p, miR-140-3p, miR-340-5p, miR-452-5p, miR-769-5p and miR-1304-p5, miR-340-5p	Esophageal squamous carcinoma	Upregulated expression of these exosomal miRNAs in hypoxic esophageal squamous carcinoma cells	[44]
lncRNA-UCA1	Ladder cancer	Promotes cell proliferation, migration, and invasion	[18]
miR-301a	Glioma	Activates Wnt/β-catenin signaling and promotes radiation resistance by targeting TCEAL7	[52]
miR-135b	Multiple myeloma	Enhances angiogenesis by targeting factor-inhibiting HIF-1	[84]
Exosomal proteins			
Cell migration-inducing and hyaluronan-binding protein (CEMIP)	Brain cancer	Increases pro-inflammatory cytokines Ptgs2, Tnf, and CCL/CXCL, which promote brain vascular remodeling and metastasis	[85]
Integrin β3	Lung Cancer	Mediates a brain-tropic metastasis pattern and may serve as a novel prognostic biomarker for brain metastasis	[75]
Complement factor H (CFH)	Hepatocellular carcinoma	Promotes tumor cell growth, migration, invasiveness, and liver tumor formation in mice	[48]
VEGF	TME	Its overexpression along with the activation of VEGFR induces immune-suppressive	[23]
Wnt5b	Pancreatic cancer	Promotes cancer cell migration and proliferation	[76]
Exosomal lipids			
Triglycerides	Prostate cancer	The activation of lipogenesis-related enzymes and signaling molecules causes increased accumulation of triglycerides in exosomes	[83]
Phosphatidylserine	TME	Externalization of phosphatidylserine from the inner to the outer membrane leaflet of cells and exosomes provides strong immune-suppressive signals	[23]
Phosphatidylserine	-	A critical molecule in the exosomal uptake by HUVECs.	[81]

**Table 2:** Observed effects of hypoxic exosomes on various immune cells.

Immune Cell	Mechanism Involved	Effects Observed	Reference
Macrophage	The transfer of let-7a miRNA resulted in the suppression of the insulin-Akt-mTOR signaling pathway	Improved macrophage recruitment and M2-like polarization in vitro and in vivo Increased expression of immunomodulators, such as CSF-1, CCL2, FTH, FTL, and TGFβ	[24]
Macrophage	miR-301a-3p activates the PTEN/PI3Kγ signaling pathway	Hypoxic exosomal miR-301a-3p induces M2 polarization of macrophages Hypoxic exosomes enhance malignant behaviors of pancreatic cancer cells	[78]
Macrophage	Macrophage expression of HIF-1α	Tumor-associated macrophages suppress tumor-infiltrating T cells	[146]
Macrophage	Exosomal miRNAs are induced by hypoxia vian HIFs	Tumor-associated macrophages educated by hypoxic exosomes derived from cancer cells promote tumor proliferation and migration in a feedback loop.	[147]
Macrophages Monocytes	MyD88-p38-STAT3 signaling The tumor cell-released TLR4-mediated autophagosomes-PD-L1 axis	Tumor cell-released autophagosomes-induced macrophage polarization into M2-like phenotype characterized by the expression of PD-L1 and IL-10 M2-like phenotype with increased expression of PD-L1, CD163, and IL-10, but decreased HLA-DR with T cell-suppressive function	[93]
Monocyte	Exosomal delivering of miRNA-21	Monocyte transformation to M2-like macrophages via miRNA-21, with increased expression of IL-10 and CD206	[98]
T-cells	miR-24-3p targets FGF11 to inhibit T-cell function	Hypoxia increases cellular and exosomal miR-24-3p levels and enhances the inhibitory effect on T-cell proliferation and differentiation	[57]
T cells	Targeted depletion or elimination of hypoxia in tumors	Increased T cells infiltration into hypoxic zones and downregulation of MDSCs	[105]
Γδ T-cell (lymphocyte)	Hypoxic exosomes regulate MDSC function in a miR-21/PTEN/ PD-L1-axis-dependent manner	There is an enhanced suppressive effect of MDSCs on γδ T cells	[108]
γδ T cells	Reduced calcium efflux and the expression of CD107a in γδT cells	Decreased antitumor cytotoxicity of γδT cells observed under hypoxia	[114]
MDSC	Increased level of exosomal S100A9 vian HIF-1α-dependent mechanism	MDSCs enhance colorectal cancer cell stemness and growth	[119]
MDSCs Regulatory B cells	Microvesicles transport membrane-bound PD-L1 from MDSCs to B cells	Suppressed CD8^+^ T-cell activation, and increased CD155, TGFβ, and IL10	[135]
DC	PD-1/PD-L1 pathway	DCs treated with tumor cells exosomes significantly increase PD-1+CD8+T cells	[130]
DC	Hypoxia induces upregulation of microRNA 21 in DCs	Decreased expressions of CD80, CD86, and MHCII on DCs	[126]
B cells	Hypoxia-induced IL-10 secretion via HMGB1	Hypoxia significantly enhances the level of HMGB1 on tumor cell-released autophagosomes leading to the induction of IL-10-producing B cells that suppress CD4+ and CD8+ T cells	[134]
B cells	IL-10-dependent manner Activation of the TLR2-MyD88-NF-κB signal pathway in B cells	B cells differentiate into IL-10-producing regulatory B cells with a distinct phenotype of CD1d(+) CD5(+), which could potently inhibit CD8(+) and CD4(+) T cell responses	[133]
NK cells	Hypoxic tumor-derived microvesicles miR-23a and TGF-β1	Transfer of TGF-β1 and miR-23a to NK cells, decreases NKG2D, thereby inhibiting NK cell function.	[100]
Mast cells	CD40L-CD40 interaction	Promote PMN-MDSCs activity and T-cell inactivity to favor the suppression of antitumor activities and encourage tumor onset	[137]
Mast cells	Increased expressions of HIF-1α, VEGF, and H1	Increased tumor growth and angiogenesis. And decreased survival rate of the mice	[139]
Mast cells	CCL5 dependent	Increased suppression of antitumor function and enhanced tumor progression	[141]

## Data Availability

Not applicable.
